# Anti-influenza A virus activity of rhein through regulating oxidative stress, TLR4, Akt, MAPK, and NF-κB signal pathways

**DOI:** 10.1371/journal.pone.0191793

**Published:** 2018-01-31

**Authors:** Qian-Wen Wang, Yun Su, Jiang-Tao Sheng, Li-Ming Gu, Ying Zhao, Xiao-Xuan Chen, Cheng Chen, Wei-Zhong Li, Kang-Sheng Li, Jian-Ping Dai

**Affiliations:** 1 Department of Microbiology and Immunology, Shantou University Medical College, Shantou, Guangdong, China; 2 Department of Veterinary Medicine, University of Maryland, College Park, Maryland, United States of America; Deutsches Primatenzentrum GmbH - Leibniz-Institut fur Primatenforschung, GERMANY

## Abstract

Rhein, an anthraquinone compound existing in many traditional herbal medicines, has anti-inflammatory, antioxidant, antitumor, antiviral, hepatoprotective, and nephroprotective activities, but its anti-influenza A virus (IAV) activity is ambiguous. In the present study, through plaque inhibition assay, time-of-addition assay, antioxidant assay, qRT-PCR, ELISA, and western blotting assays, we investigated the anti-IAV effect and mechanism of action of rhein *in vitro* and *in vivo*. The results showed that rhein could significantly inhibit IAV adsorption and replication, decrease IAV-induced oxidative stress, activations of TLR4, Akt, p38, JNK MAPK, and NF-κB pathways, and production of inflammatory cytokines and matrix metalloproteinases *in vitro*. Oxidant H_2_O_2_ and agonists of TLR4, Akt, p38/JNK and IKK/NF-κB could significantly antagonize the inhibitory effects of rhein on IAV-induced cytopathic effect (CPE) and IAV replication. Through an *in vivo* test in mice, we also found that rhein could significantly improve the survival rate, lung index, pulmonary cytokines, and pulmonary histopathological changes. Rhein also significantly decreased pulmonary viral load at a high dose. In conclusion, rhein can inhibit IAV adsorption and replication, and the mechanism of action to inhibit IAV replication may be due to its ability to suppress IAV-induced oxidative stress and activations of TLR4, Akt, p38, JNK MAPK, and NF-κB signal pathways.

## Introduction

Influenza A virus (IAV) is a highly transmissible respiratory pathogen that can cause seasonal flu epidemics and periodic worldwide pandemics. During the 2009 pandemic (H1N1) in the USA, about 43–89 million people became ill, and approximate 8,870–18,300 people died due to the 2009 pandemic H1N1 infection [1]. Though vaccination is the best strategy to fight IAV infection, IAV vaccine production is currently still not satisfied due to the continuous evolution of IAV major antigens. In addition, classical anti-IAV drugs, such as M2 channel blockers (e.g. amantadine and rimantadine) and neuraminidase inhibitors (e.g. oseltamivir and zanamivir), are limited in use by their side effects and the continual emergence of resistant viral strains. So, the development of novel anti-IAV drugs continues to be urgent [2].

Phytotherapy has a long history in China, Korea, Japan, and India. In the past hundreds of years, traditional medicine has played an important role in fighting IAV infection. Now, research of antiviral drugs from herbs and other natural resources with traditionally long-confirmed effects is believed to be an efficient approach, which is expected to result in finding new and effective drugs. In our previous researches, we have set up several high-throughput screening (HTS) method and screened hundreds of traditional Chinese medicines (TCM), and found that the crude extract of rhubarb (*Rheum palmatum* L.) possesses anti-IAV activity *in vitro* [3–5]. In fact, Lin TJ *et al* have also found that the ethanolic extract of rhubarb can inhibit IAV (H1N1) infection [6]. The major active ingredient of the crude extract of rhubarb is anthraquinone that includes several compounds, such as rhein, emodin, chrysophanol, physcion, and aloe-emodin. Among them, rhein is also extensively found in several other TCMs, such as *Aloe barbadensis* Miller, *Cassia angustifolia* Vahl., and *Polygonum multiflorum* Thunb. Rhein has two hydroxide radicals and one carboxyl with very strong chemical polarity and is thought to possess high electrochemical oxidoreduction activity [7]. Now, it is well known that rhein has antioxidant, antiviral, anti-inflammatory, antitumor, anti-fibrosis, hepatoprotective, and nephroprotective activities [8]. However, the anti-IAV effect of rhein is still ambiguous.

Pathogenesis of influenza in humans is actually caused by a combination of viral direct damage and host immune injury. Excessive host immune response is harmful and often results in severe immunologic injury. Infection of highly pathogenic IAV usually causes substantial immunopathology and leads to acute lung injury (ALI) and acute respiratory distress syndrome (ARDS) with substantial morbidity and mortality. It has been reported that IAV infection can lead to the activation of toll-like receptor (TLR) signaling pathways. Lasting activation of TLR3 is proved to be harmful for IAV-induced acute pneumonia [9, 10]. Activation of TLR4 can determine IAV entry and tropism via MyD88 expression and p38 MAPK activation [11]. Inactivated H5N1 avian IAV can induce oxidative stress and ALI through the TLR4-TRIF- TRAF6-NF-κB signaling pathway [12]. Especially, activations of PI3K/Akt, MAPK, and NF-κB pathways are proved to be required or even support IAV replication, and are crucial in the development of ALI [13–19]. In the present study, based on our previous HTS assays, we have examined the anti-IAV effect of rhein *in vitro* and *in vivo* and investigated the mechanism of action of rhein, mainly focusing on the TLR, PI3K/Akt, MAPK and NF-κB signaling pathways.

## Materials and methods

### Materials

Rhein (C_15_H_8_O_6_, purity > 98%, ^#^110757) was purchased from Chinese Materials Research Center, National Institute for the Control of Pharmaceutical and Biological Products (Beijing, China). DMSO, Tosylsulfonyl phenylalanyl chloromethyl ketone (TPCK)- trypsin (^#^4370285-1KT), ribavirin (^#^R9644-10MG) and sulforhodamine B (SRB, ^#^230162-5G) were purchased from Sigma-Aldrich, Inc (St. Louis, MO, USA). LAM-MS (^#^tlrl-lams), Poly(I:C) (^#^tlrl-pic), LPS-B5 (^#^tlrl-pb5lps) were purchased from InvivoGen (San Diego, California, USA). EGF (^#^8916), anisomycin (^#^2222) and antibodies for human ERK1/2 (^#^8867), p-ERK1/2 (^#^13148), p-JNK (^#^3708), JNK(^#^4671), p-p38(^#^4092), p38(^#^14451), p65 (^#^4764) and β-actin (^#^12262) proteins were bought from Cell Signaling Technology® Inc Company (Danvers, MA, USA). Antibodies for human TLR2 (sc-21760), TLR3 (sc-517367), TLR4 (sc-293072), Lamin B1(sc-56144) proteins and secondary horseradish peroxidase- conjugated anti-rabbit, anti-mouse or anti-goat IgG were acquired from Santa Cruz Biotechnology (Santa Cruz, CA, USA). All other chemicals and solvents were commercially available and of analytical grade. Rhein was dissolved in DMSO as stock solution, and diluted with Dulbecco’s modified Eagle medium (DMEM, Invitrogen, Carlsbad, CA, USA) when used.

### Cells, viruses, and cytotoxicity assay

Madin-Darby canine kidney (MDCK) cells and A549 lung cancer cells were cultured in DMEM medium and incubated in a 5% CO_2_ humidified incubator. Virus stocks of IAV subtypes A/ShanTou/169/06 (ST169, H1N1) and A/PuertoRico/8/34 (PR8, H1N1) were prepared in MDCK cells. Virus titer was determined by a plaque formation assay [4]. The cytotoxicity of rhein on MDCK and A549 cells was determined using a MTT assay [20]. The concentration of rhein required to lower cell viability by 50% (CC_50_) was calculated using Origin 8.0 software. The highest concentration that did not show significant cytotoxicity was used as the test concentration *in vitro*. The experiment was repeated five times and each experimental condition was performed in triplicate (n = 5). All experiments with IAV were performed in the biosafety level 3 laboratory.

### Plaque formation, plaque inhibition, and time-of-addition assays

The viral titers of the supernatants were determined by plaque formation assay as our previous report [5]. Plaque inhibition assay was also performed as previously reported [5]. Briefly, A549 cells were incubated with virus growth medium (VGM, DMEM containing 2.5 μg/mL TPCK-trypsin and 3.2% bovine serum albumin (BSA)) that contained IAV (MOI = 0.001) and different concentrations of rhein for 1 h, after washing with PBS 3 times, VGM medium with or without rhein was added. After 48 h, the supernatant was collected and the viral titer was determined by a plaque formation assay. The time-of-addition assay was performed as our previous report [4], which contained four tests: (a) direct inactivation assay: before infection, IAV virion was incubated with a VGM medium containing rhein (10 μg/mL), after 3 h, IAV virion was gathered by ultra-filtration and washed with PBS 3 times, then was used to infect MDCK cells and the cells were further cultured for 12 h; (b) influence-on-cell assay: before infection, MDCK cells were incubated with VGM medium containing rhein (10 μg/mL), after 3 h, the cells were washed with PBS 3 times, infected with normal IAV and further cultured for 12 h; (c) influence-on-viral adsorption assay: during viral adsorption, rhein (10 μg/mL) was added, after adsorption for 1 h, the cells were washed with PBS 3 times and cultured with normal VGM medium for 12 h; and (d) different-time-points post infection (p.i.) assay: after IAV infection, rhein (10 μg/mL) was added at 1, 2, 3, 4, 5, 6, 7 and 8 h p.i., respectively, then the cells were further cultured to 12 h p.i.. MOI = 2.0. At 12 h p.i., the supernatants were gathered and the viral titer was determined by a plaque formation assay. The experiment was repeated five times and each experimental condition was performed in triplicate (n = 5).

### Antiviral assay by the sulforhodamine B (SRB) method using CPE reduction

The stock solution of IAV was diluted with VGM medium in 10-fold serial dilutions, after incubation with MDCK cells for 48 h, the TCID_50_ was calculated following the method of Reed and Muench. Antiviral activity of rhein was also evaluated by the SRB method using CPE reduction as previously reported [2, 21]. Briefly, MDCK cells were seeded in 96-well plate. 0.09 mL of virus suspension (50× TCID_50_) and 0.01 mL VGM medium containing rhein (100 μg/mL) with or without different agonists were added. At 48 h, after washing, 100 μL -20°C 70% acetone was added. After removing acetone, the plates were dried and added 100 μL 0.4% (w/v) SRB. After 1h, the plates were washed, dried and added 100 μL 10 mM Tris-base solution. OD was read at 562nm. Three wells were used each for the negative (virus-infected non-drug-treated) and mock (non-infected non-drug-treated) controls. 0.5% DMSO was used in each group. The percent protection of rhein, which is positively related to the finally remained cell viability, was calculated as the following:

Protectionoftestcompound(%)=ODtest¯ODmock¯×100% (1)

When the concentration of test compound was zero, it was the negative control (virus-infected non-drug-treated), and the results of all drug-treated groups were statistically compared with that of the negative control. Concentration of 50% protection was defined as EC_50_. Antiviral index (AI) was defined as CC_50_/EC_50_. The experiment was repeated five times and each experimental condition was performed in triplicate (n = 5).

### Antioxidant assay

The production of reduced glutathione (GSH), malondialdehyde (MDA), nitric oxide (NO), reactive oxygen species (ROS), and the activities of total superoxide dismutase (T-SOD), glutathione reductase (GR), catalase (CAT) and glutathione peroxidase (GSH-Px) were determined using commercially available kits (Jiancheng Bioengineering Institute, Nanjing, China). Briefly, A549 cells were infected with IAV (ST169, MOI = 0.001) and treated with ribavirin (25 μg/mL) and rhein (10 μg/mL), respectively. After incubation 48 h, the cell lysates were gathered, the protein levels were measured, and after centrifuged at 1000g for 10min at -4°C, the supernatants of cell lysates were used in the antioxidant assays following the manufacturer’s protocol. A549 cells that not infected, or infected but not-treated with any drugs were used as the blank control and negative control, respectively. The experiment was repeated five times and each experimental condition was performed in triplicate (n = 5).

### Quantitative real-time RT-PCR (qRT-PCR)

A549 cells were treated as aforementioned in “Antioxidant assay”. Total RNA was extracted using Trizol® Plus RNA purification kit (Invitrogen). DNA contamination in the total RNA was deleted with the addition of DNase I (Invitrogen). Total RNA was eluted in nuclease-free water and quantified spectrophotometrically at 260nm and 280nm. The qRT-PCR was performed in a 20 μL reaction mixtures containing forward and reverse primers (50 nM each), 1× SYBR green master mix (Invitrogen) and various templates. The results were presented in 2^-ΔΔCt^. The experiment was repeated five times and each experimental condition was performed in triplicate (n = 5). Primers were listed in S1 Table [4, 21].

### ELISA assay

A549 cells were treated as aforementioned in “Antioxidant assay”. Cytokines and MMPs were quantified by using specific ELISA kits following the manufacturer’s instructions. IL-1β (DKW12-3012/-2012), IL-6 (DKW12-1060/-2060), IL-8 (DKW12-1080), TNF-α (DKW12-1720/-2720) and IL-10 (DKW12-1100/-2100) ELISA Kits were purchased from Dakewe biological technology co., LTD (Beijing, China). MMP2 (ab100606), MMP3 (ab189572), MMP9 (ab100610), MMP13 (ab100605) and TIMP1 (ab100651) ELISA kits were purchased from Abcam Company (Cambridge, UK). The experiment was repeated five times and each experimental condition was performed in triplicate (n = 5).

### Western blotting assay

Western blotting assay was carried out as previously reported [5]. Proteins were extracted by using RIPA lysis buffer (Biocolor BioScience and Technology, China) according to the manufacturer’s directions. To detect NF-κB p65, nucleic protein was extracted. Protein concentration was determined by BCA assay (Thermo Scientific, Rockford, IL). Approximately 40 μg of protein extracts was separated by 8–12% SDS-PAGE and electrophoretically transferred onto polyvinylidene fluoride membranes (Millipore, Bedford, USA). After blocking with 5% non-fat dry milk in Tris-buffered saline, membranes were incubated overnight with primary antibodies, including TLR2, TLR3, TLR4, p-Akt, Akt, ERK1/2, p-ERK1/2, p-JNK, JNK, p-p38, p38, p65, lamin B1, and β-actin antibodies. Subsequently, a secondary horseradish peroxidase- conjugated anti-rabbit, anti-mouse, or anti-goat IgG antibody was applied, and then specific bands were visualized using the ECL detection kit (Thermo Fisher Scientific™, Cleveland, OH, USA). β-actin was used as a control for total proteins and lamin B1 was used as the control for nuclear proteins. Protein bands intensities were analyzed by Quantity One software (Bio-Rad Laboratories, Hercules, USA). The experiment was repeated five times (n = 5).

### *In vivo* study

This study was carried out in strict accordance with the recommendations of the Guide for the Care and Use of Laboratory Animals of the National Institutes of Health, following the *Animal Research*: *Reporting of In Vivo Experiments* (ARRIVE) *guidelines* [22, 23]. The protocol was approved by the Committee on the Ethics of Animal Experiments of the University of Shantou University (Permit Number: SUMC2017-083). All experimental operations were performed under ketamine anesthesia and all efforts were made to minimize suffering. The experiments with IAV infection were performed in a biosafety level 3 laboratory. The duration of the experiment was 15 days after IAV infection. And finally, all mice were euthanized by cervical dislocation.

Male and female SPF C57BL/6J mice (20± 2g) at 6–8 weeks old were purchased from Shanghai slack laboratory animal co., LTD (Shanghai, China). Animals were housed for 5 days for acclimation with 12-hour light-dark cycles and maintained with standard pellet feed and water *ad libitum*. The 50% mouse lethal dose (MLD_50_) was calculated by the method of Reed and Muench through a preliminary test. During experiment, 80 mice were randomly divided into 5 groups (n = 16) using the random number table and anesthetized by intraperitoneal injection of ketamine (100 mg/kg). In each group, there were half of male and female mice.

- In blank control (BC, n = 16), mice were not infected with IAV (PR8) virus but shammed with VGM medium in a 50 μL volume intranasally, and then treated with DMSO (0.5%) by oral gavage.
- In negative control (NC, n = 16), mice were infected intranasally with 10× MLD_50_ of IAV (PR8) virus in a 50 μL volume, and treated with DMSO (0.5%) by oral gavage.
- In positive control (PC, n = 16), mice were infected intranasally with 10× MLD_50_ of IAV (PR8) virus in a 50 μL volume, and treated with oseltamivir (10 mg/kg/day) by oral gavage.
- In rhein-treated groups (Rhe75 and Rhe150, n = 16, respectively), mice were infected intranasally with 10× MLD_50_ of IAV (PR8) virus in a 50 μL volume, and treated with rhein (75 mg/kg/day and 150 mg/kg/day, respectively) by oral gavage. The test doses are determined according to the previous reports [24–26] and our preliminary test.

DMSO (0.5%), oseltamivir and rhein were given twice a day (at a 12-hour interval) for 6 days, starting 24 h after randomly grouping and before virus exposure. The body weights and survivals of ten mice in each group (n = 10) were monitored daily for 14 days after virus exposure. For humane endpoint, animals were immediately euthanized when their weights reduced 30% and displayed with obvious ruffled fur and reduced mobility. At day 6 p.i., another six mice in each group (n = 6) were euthanized. The lung index was assessed by determining the percent of lung wet weight (g) to body weight (g) (lung index = lung wet weight (g) ÷ body weight (g) × 100%). The collected lungs were further separated into two sets, right lungs were fixed in 10% formalin and left lungs were frozen at -80°C. To determine the viral load and cytokines in lungs, left lungs were homogenized in 1 ml of cold DMEM medium and the total protein levels were measured. Viral load and cytokines was determined by TCID_50_ assay and ELISA assay, respectively. The unit was corrected according to the amount of total protein. To examine pathological changes, right lungs were embedded in paraffin, sectioned at 5 μm for haematoxylin and eosin (H&E) staining, and examined at 200× and 400× magnifications. Each slide was assessed by two separate investigators in a blinded manner.

### Statistical analysis

Data were presented as mean ± SD and analyzed using SPSS13.0 software. The significant differences between groups were assessed by Student’s t-test, one-way ANOVA with *post hoc* Dunnett’s test, Kruskal-Wallis H test or Log rank and Breslow tests. Results were considered statistically different when the *P* values were equal to or less than 0.05.

## Results

### Rhein inhibited the replication of IAV *in vitro*

**Fig 1:**
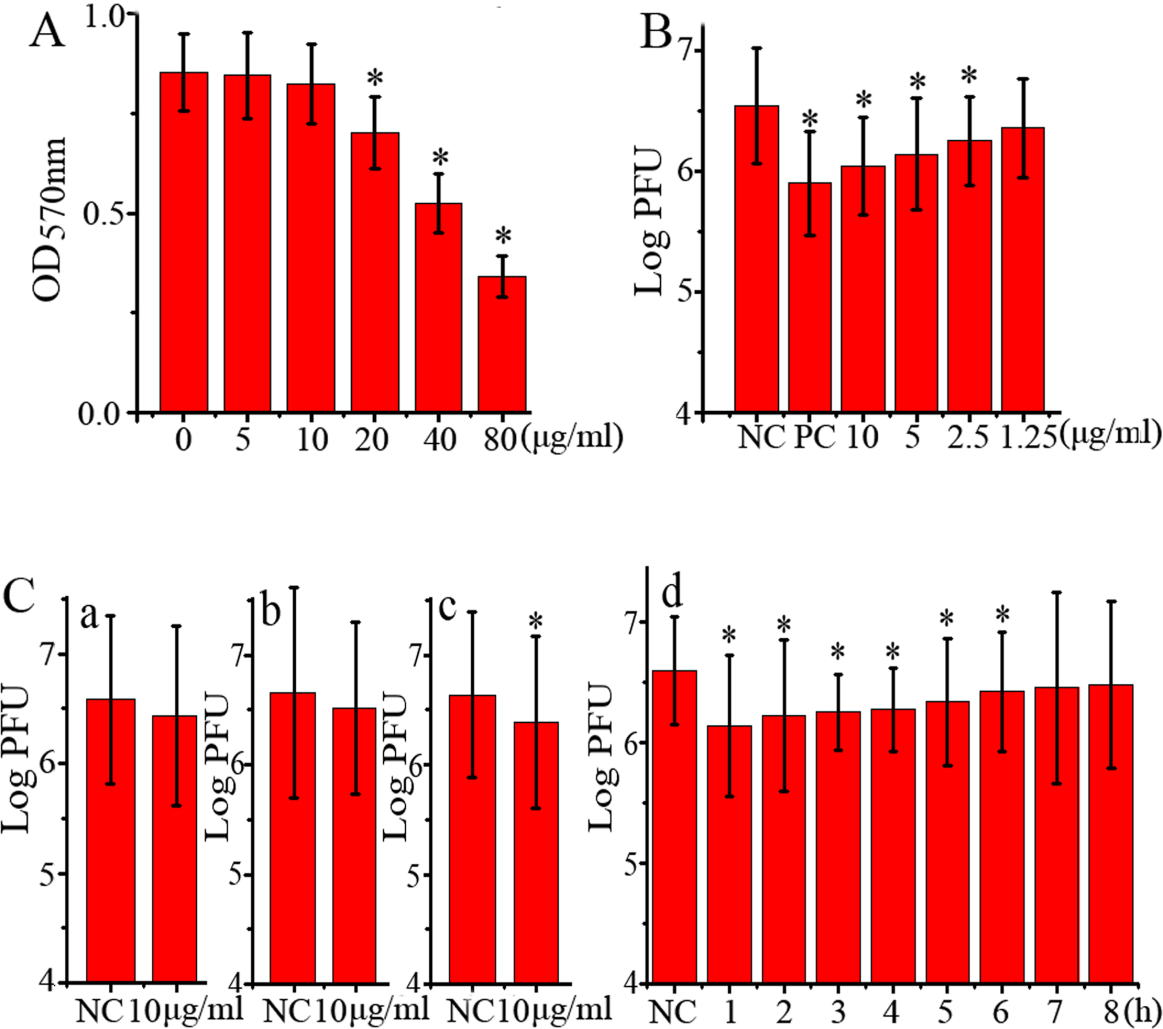
Anti-IAV activity of rhein *in vitro*. (A) The cytotoxicity of rhein was determined by a MTT method, *P < 0.05 *vs*. the blank control (0 μg/mL). (B) Inhibition of rhein on IAV (ST169, H1N1) replication was determined by a plaque inhibition assay. In the negative control (NC), MDCK cells were infected with IAV (MOI = 0.001) but not treated with any drugs; in the positive control (PC) and rhein-treated groups, MDCK cells were infected (MOI = 0.001) and treated with ribavirin (25 μg/mL) and rhein (1.25, 2.5, 5, and 10 μg/mL), respectively. After 48 h p.i., the supernatants were harvested and the titers were determined by a plaque formation assay. (C) The results of the time-of-addition assay, which contained four tests: (a) direct inactivation assay, (b) influence-on-cell assay, (c) influence-on-viral adsorption assay, and (d) different-time-points p.i. assay. MOI = 2.0. 0.5% DMSO was used as the negative control (NC). After 12 h p.i., the supernatants were harvested and the viral titer was determined by a plaque formation assay. The experiment was repeated five times and each experimental condition was performed in triplicate (n = 5). All data shown were mean ± SD. *P < 0.05 *vs*. the NC group.

As showed in Fig 1A, the CC_50_ of rhein in A549 cells was 64.59 μg/mL (Y = -0.1889 LnX + 1.2137, R^2^ = 0.9377). At the concentration of 10 μg/mL, rhein did not show significant cytotoxicity and we chose this concentration (10 μg/mL) as the test concentration in the following pharmacological experiments.

Through a plaque inhibition assay, we found that rhein could significantly inhibit the proliferation of IAV (ST169, H1N1) in the concentration range from 2.5 to 10 μg/mL, the EC_50_ was 1.51 μg/mL (Y = -0.1544 LnX + 6.3961, R^2^ = 0.9998) and the AI was 42.77 (Fig 1B).

In addition, through a time-of-addition assay, we further found that rhein could not directly inactivate IAV (Fig 1C a) and had no significant influence on cells (Fig 1C b), but could inhibit IAV adsorption (Fig 1C c) and replication when rhein was added at 1h and up to 6h p.i. (Fig 1C d).

### Rhein could improve IAV-induced oxidative stress *in vitro*

Rhein has been reported to possess antioxidant activity [24, 27, 28]. In the present study, we examined the effect of rhein on IAV-induced oxidative stress in A549 cells at 48 h p.i.. As showed in Table 1, IAV infection (NC group) could significantly increase the production of MDA, NO, and ROS, significantly decrease the level of GSH, and inhibit the activities of T-SOD, GR, CAT, and GSH-PX, comparing with the BC group. Rhein could significantly decrease the concentration of MDA, NO, and ROS, increase the level of GSH, and up-regulate the activities of T-SOD, GR, CAT, and GSH-PX, comparing with the NC group. Ribavirin (25 μg/mL) almost had no significant effect on IAV-induced oxidative stress.

**Table 1:** Effect of rhein on IAV-induced oxidant stress.

Group	GSH	MDA	NO	ROS
BC	31.65±2.66	9.87±0.9	34.62±3.95	1.00±0.00
NC	13.57±1.65^#^	22.21±1.41^#^	73.56±8.87^#^	2.32±0.26^#^
PC	16.63±2.05	15.57±1.16*	65.78±6.64	2.01±0.25
Rhe	21.32±4.42*	13.28±1.15*	45.65±4.67*	1.51±0.17*
Group	T-SOD	GR	CAT	GSH-Px
BC	72.56±7.55	91.54±9.05	62.54±6.03	4.21±0.53
NC	33.01±5.31^#^	39.86±4.65^#^	21.05±3.87^#^	2.22±0.43^#^
PC	44.46±5.37	50.53±8.99*	31.67±4.37	3.29±0.62
Rhe	62.17±6.74*	81.78±9.39*	46.37±4.95*	3.71±0.53*

In the blank control (BC), A549 cells were not infected by IAV (ST169) and treated with 0.5% DMSO. In the negative control (NC), positive control (PC) and rhein-treated groups, A549 cells were infected with IAV (MOI = 0.001) and treated with 0.5% DMSO, ribavirin (25 μg/mL), and rhein (10 μg/mL), respectively. After 48 h p.i., the cell lysates were harvested. The levels of GSH (nmol/mg protein), MDA (nmol/mg protein), NO (nmol/mg protein), ROS (fold change of fluorescence values to the BG group), T-SOD (U/mg protein), GR (nmol NADPH oxidized/min/ mg protein), CAT (nmol H_2_O_2_ /min/mg protein), and GSH-Px (mU/mg protein) were determined using commercially available kits. The experiment was repeated five times and each experimental condition was performed in triplicate (n = 5). All data shown were mean ± SD.

^#^*P* < 0.05 *vs*. the BC group

*P < 0.05 *vs*. the NC group.

### Rhein could inhibit IAV-induced activations of TLR2, TLR3, TLR4, Akt, p38, JNK MAPK and NF-κB pathways *in vitro*

**Fig 2:**
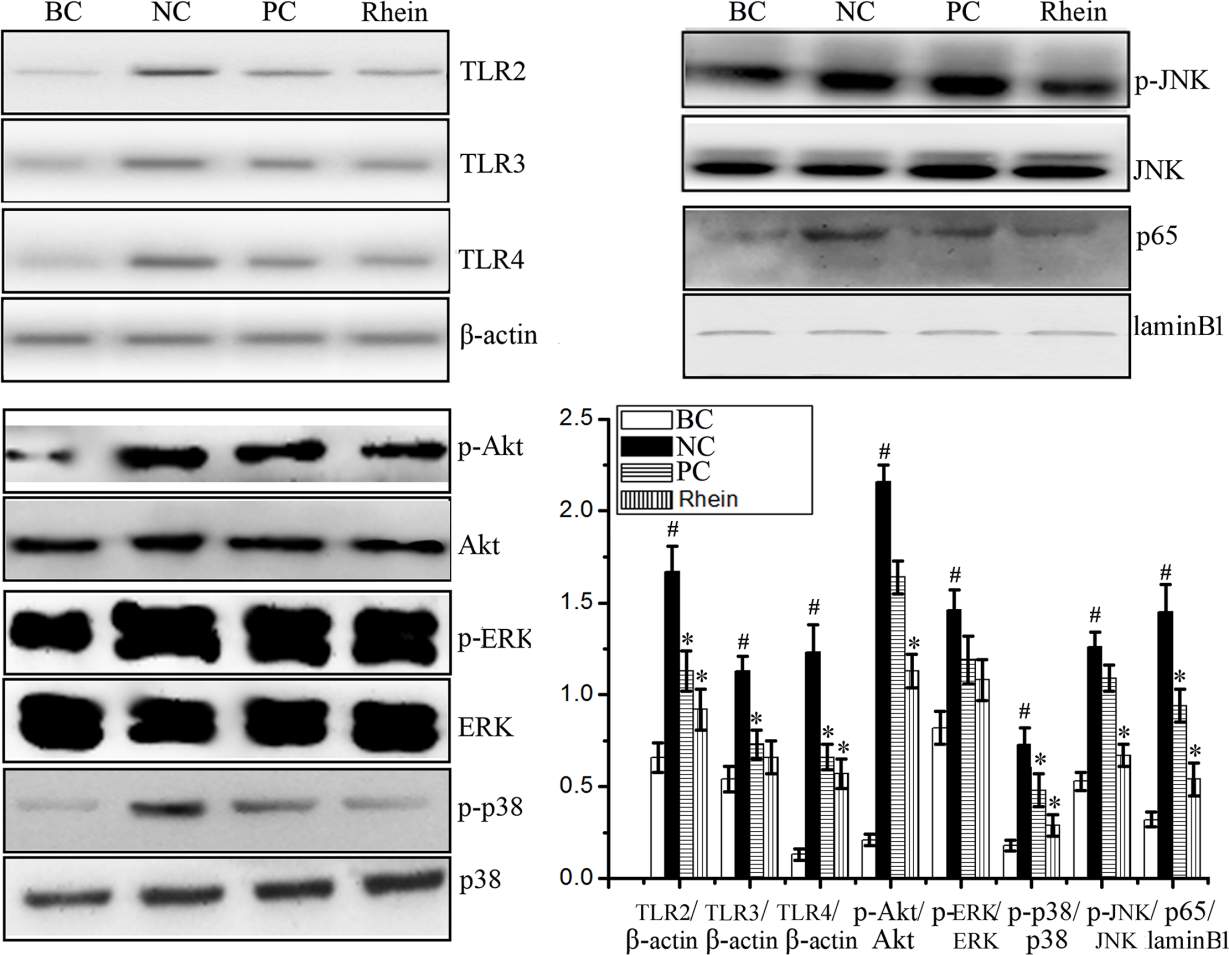
Effects of rhein on TLRs, Akt, MAPK and NF-κB signaling pathways after IAV infection. The treatment of the blank control (BC), negative control (NC), positive control (PC), and rhein-treated group (rhein) was same with that of the “antioxidant assay”. After 48 h p.i., the cells were harvested. The expressions of TLR2, TLR3, and TLR4, the phosphorylations of Akt, ERK, p38, JNK MAPK, and the nuclear translocation of NF-κB p65 were determined by western blotting assay. The experiment was repeated five times (n = 5). All data shown were mean ± SD. ^#^*P* < 0.05 *vs*. the BC group, *P < 0.05 *vs*. the NC group.

As showed in Fig 2, IAV infection could up-regulate the expressions of TLR2, TLR3, and TLR4, phosphorylations of Akt, p38, JNK, ERK MAPK and nuclear translocation of NF-κB p65 protein. Rhein could significantly decrease IAV-induced expressions of TLR2, TLR3, and TLR4, significantly inhibit IAV-induced phosphorylations of Akt, p38, JNK MAPK and nuclear translocation of NF-κB p65, but not for the phosphorylation of ERK MAPK. Ribavirin, which is a purine nucleoside analogues, also could significantly reduce IAV-induced expressions of TLR2, TLR3, and TLR4, phosphorylation of p38 MAPK and nuclear translocation of NF-κB p65, but not for the phosphorylations of Akt, JNK, and ERK MAPK.

### The counteracting effects of the agonists of the signal pathways on the antiviral activity of rhein

**Fig 3:**
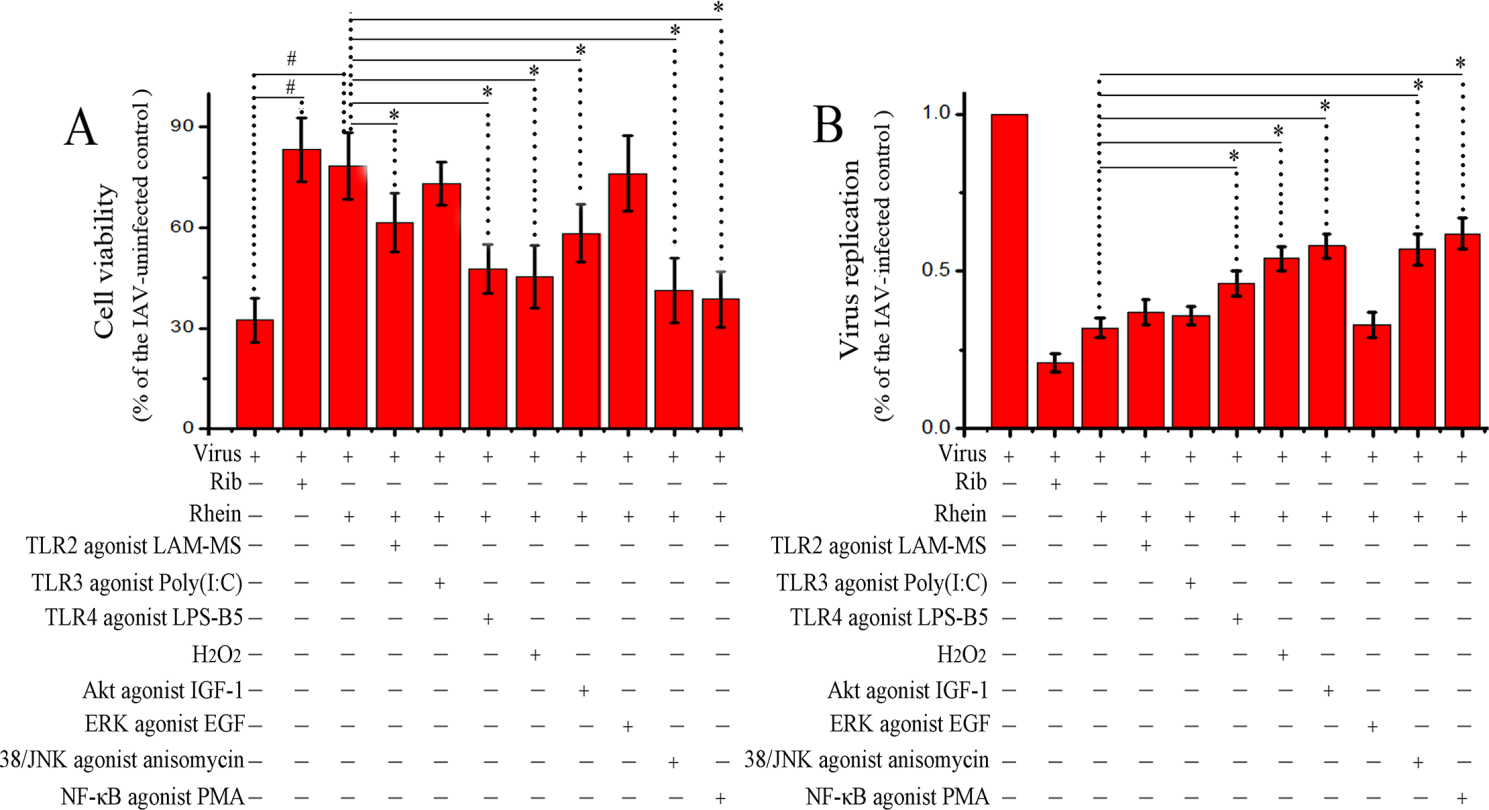
Counteracting effects of different agonists on the antiviral activity of rhein. A549 cells were infected with IAV (MOI = 0.001) and treated with or without ribavirin (Rib, 25 μg/mL), rhein (10 μg/mL), TLR2 agonist LAM-MS (10 μg/ml), TLR3 agonist Poly(I:C) (10 μg/ml), TLR4 agonist LPS-B5 (1 μg/ml), oxidant H_2_O_2_ (100 μM), Akt agonist IGF-1 (IGF-1 100 ng/ml), ERK agonist EGF (100 ng/ml), p38/JNK agonist anisomycin (10 μM), and NF-κB agonist PMA (1 μg/ml). Ater 48 h p.i., the antiviral activity was determined by a SRB method (A), IAV replication was determined by a qRT-PCR assay (B). The experiment was repeated five times and each experimental condition was performed in triplicate (n = 5). All data shown were mean ± SD. ^#^ *P* < 0.05 *vs*. the only virus-infected control, * *P* < 0.05 *vs*. the virus + rhein control.

As aforementioned, rhein could significantly decrease IAV-induced activations of TLR2, TLR3, TLR4, Akt, p38, JNK MAPK, and NF-κB pathways, we further examined the counteracting effects of the agonists of these signal pathways on the antiviral activity of rhein. We first examined the counteracting effect by a SRB method. As showed in Fig 3A, IAV infection could significantly decrease the cell viability, while ribvirin and rhein could significantly inhibit IAV-induced decrease of cell viability. As for the effects of the agonists, comparing with the IAV-infected rhein-treated group, TLR2 agonist (LAM-MS), TLR4 agonist (LPS-B5), oxidant (H_2_O_2_), Akt agonist (IGF-1), p38/JNK agonist (anisomycin), and IKK/NF-κB agonist (PMA) could significantly counteract the action of rhein, but TLR3 agonist (Poly(I:C)) and ERK agonist (EGF) had not significant effect. In addition, we also determined the replication of IAV by a qRT-PCR assay. As showed in Fig 3B, only TLR4 agonist, H_2_O_2_, Akt agonist, p38/JNK agonist, and IKK/NF-κB agonist could significantly antagonize the inhibition of rhein on IAV replication, while other agonists had no significant effect.

### Rhein regulated IAV-induced production of cytokines *in vitro*

**Fig 4:**
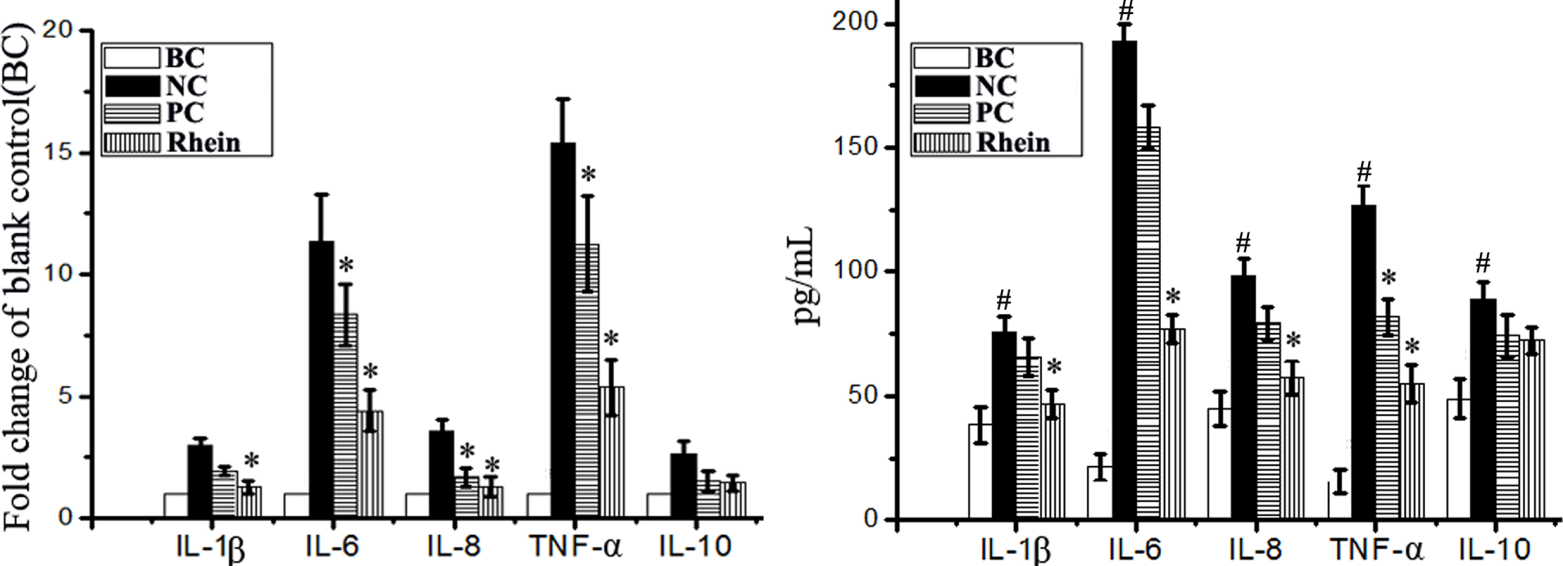
**Effect of rhein on the production of cytokines after IAV infection determined by qRT-PCR (A) and ELISA (B) assays**. The treatment of the blank control (BC), negative control (NC), positive control (PC), and rhein-treated group (rhein) was same with that of the “antioxidant assay”. The experiment was repeated five times and each experimental condition was performed in triplicate (n = 5). All data shown were mean ± SD. ^#^*P* < 0.05 *vs*. the BC group, *P < 0.05 *vs*. the NC group.

IAV-induced increase of inflammatory cytokines is an important pathological factor in IAV-related ALI and ARDS. As showed in Fig 4, under the stimulus of IAV infection, the expressions of IL-1β, IL-6, IL-8, and TNF-α were significantly increased, but the expression of IL-10 was not significant. Rhein could significantly reduce the expressions of IL-1β, IL-6, IL-8, and TNF-α, but not for the expression of IL-10, comparing with the NC control.

### Rhein could inhibit IAV-induced production of matrix metalloproteinases

**Fig 5:**
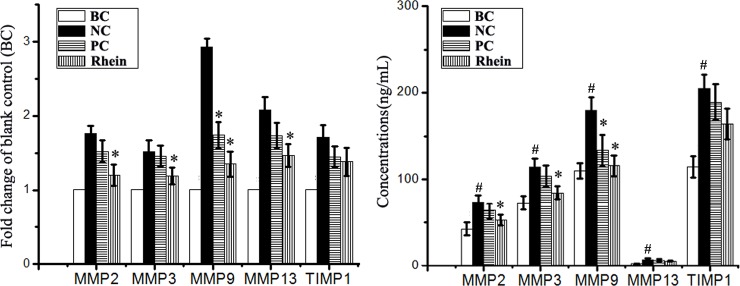
**Effect of rhein on the production of MMPs after IAV infection determined by qRT-PCR (A) and ELISA (B) assays.** The treatment of the blank control (BC), negative control (NC), positive control (PC), and rhein-treated groups (Rhein) was same with that of the “antioxidant assay”. The experiment was repeated five times and each experimental condition was performed in triplicate (n = 5). All data shown were mean ± SD. ^#^*P* < 0.05 *vs*. the BC group, *P < 0.05 *vs*. the NC group.

Matrix metalloproteinase (MMP) also plays an important role in the pathogenesis of ALI and ARDS [29, 30]. As showed in Fig 5, IAV infection (NC group) could significantly up-regulate the expressions of MMP2, MMP3, MMP9, MMP13, and TIMP-1, comparing with the BC group. Rhein could significantly inhibit the mRNA transcriptions of MMP2, MMP3, MMP9, and MMP13, but not for the transcription of TIMP-1. At the protein level, rhein could significantly inhibit the production of MMP2, MMP3, and MMP9, but not for the production of MMP13 and TIMP-1, comparing with the NC group.

### Rhein improved IAV-induced pulmonary inflammation and histopathological changes *in vivo*

**Fig 6:**
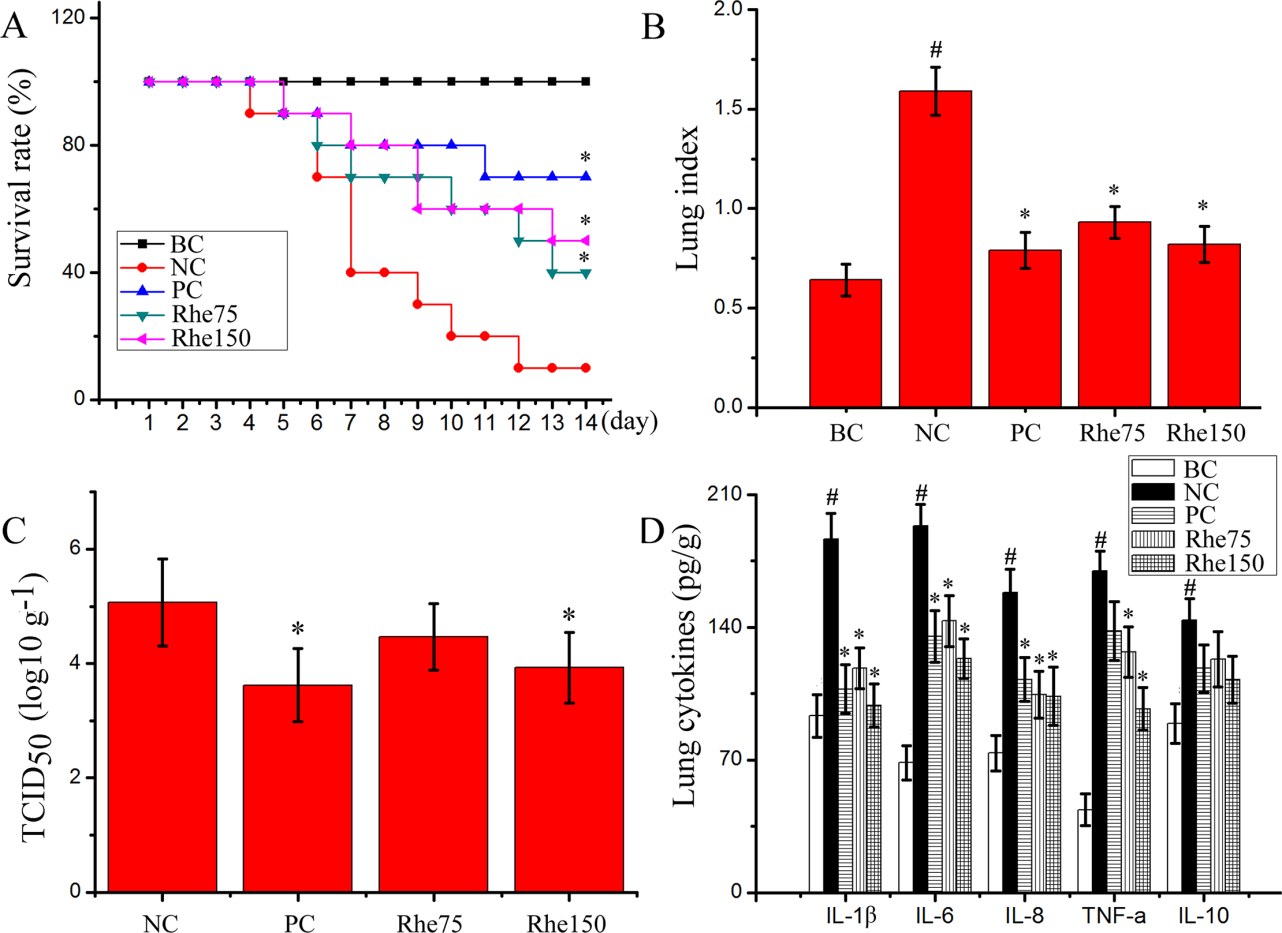
Anti-IAV activity of rhein in mice. In the blank control (BC), mice were not infected with IAV (PR8) but shammed with VGM medium and treated with DMSO (0.5%). In the negative control (NC), positive control (PC), and rhein-treated groups (Rhe75 and Rhe150), mice were infected with 10× MLD_50_ of IAV (PR8) and treated with DMSO (0.5%), oseltamivir (10 mg/kg/day) and rhein (75 mg/kg/day and 150 mg/kg/day), respectively. (A) The survival rate was observed for 14 days and analyzed by using Kaplan-Meier analysis with Log-rank and Breslow tests. (B) The lung index was assessed by determining the percent of lung wet weight (g) to body weight (g) (lung index = lung wet weight (g) ÷ body weight (g) × 100%). (C and D) The pulmonary viral load and cytokines were determined by TCID_50_ and ELISA assays, respectively. Data were mean ± SD. Ten mice were used in the survival rate assay (n = 10) and six mice were used in the lung index, pulmonary viral load, and pulmonary cytokines assays (n = 6). ^#^*P* < 0.05 *vs*. the BC group, *P < 0.05 *vs*. the NC group.

**Fig 7:**
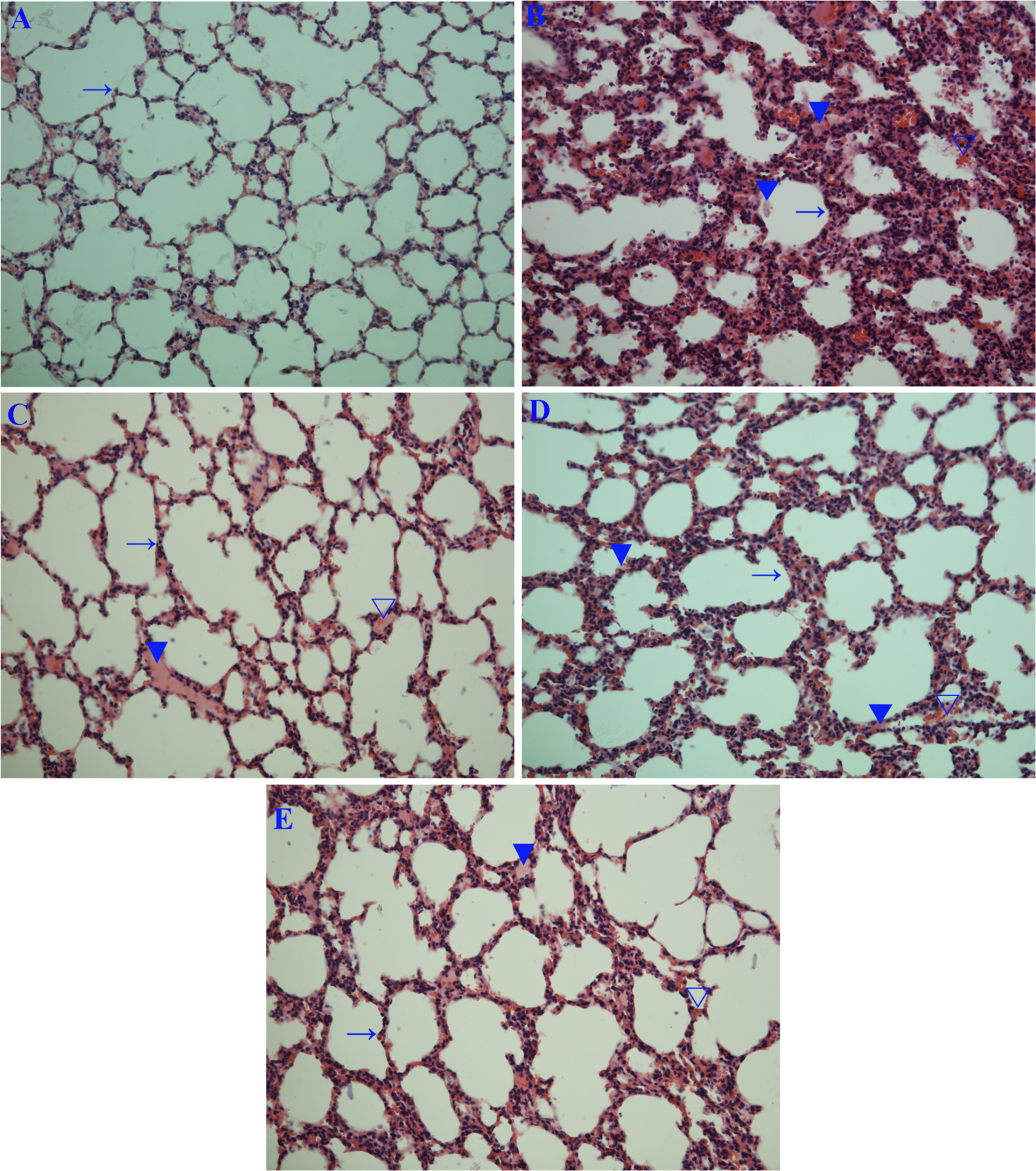
Influence of rhein on the histopathological changes. Mice were treated as mentioned in **Fig 6**. At day 6 p.i., six mice from each group were sacrificed. The right lungs were used for H&E staining assay. (A) Blank control (BC), (B) Negative control (NC), (C) Positive control (PC), (D and E) Rhein-treated groups (Rhe75 and Rhe150, respectively). (→) alveolar wall, (▼) inflammatory exudation, (▽) hemorrhage (erythrocytes). The original magnification was 200×.

Finally, we also determined the influence of rhein on influenzal virus pneumonia in mice. As showed in Fig 6, rhein could significantly improve the survival rate of mice infected with IAV (PR8), significantly reduce the lung index and lung cytokines IL-1β, IL-6, IL-8, and TNF-α. Rhein also significantly reduced pulmonary viral load at a high dose (150 mg/kg/day) determined by a TCID_50_ assay at 6 days p.i.. Additionally, rhein also improved IAV-induced pulmonary histopathological changes, decreasing alveolar exudation, the destruction of alveolar wall, and alveolar hemorrhage caused by IAV infection (Fig 7).

## Discussion

Traditional medicine is an important means of healthcare for up to 80% of the population in Asian and African countries [31]. In recent years, the popularity for use of traditional medicine as complementary medication in western countries has risen dramatically. In the United States, the use of herbal products is estimated at between 20–38% among adults, while in some European countries, this estimate rises to a range of 50–70% [32–34]. Now, traditional medicine has been getting more and more public attention and the research of new drugs from traditional medicines with long-confirmed effects may be an efficient strategy. Armed with this idea, we have previously screened many traditional herbal medicines and many compounds from herbal medicines have been identified to possess anti-IAV activity, but their mechanisms of action are not very well understood. In the present study, we have investigated the effect and mechanism of action of rhein against IAV infection.

Lin TJ *et al* have reported that the crude extract of rhubarb (*Rheum tanguticum*) can reduce IAV yields at -3 to 9 h, -3 to -1 h, -1 to 0 h, 0 to 9 h, 3 to 9 h, and 6 to 9 h p.i., and concluded that the crude extract of rhubarb can inhibit IAV via a variety of different mechanisms, including direct inactivation (-3 to -1 h p.i.), inhibition of viral adsorption (-1 to 0 h), and interferences of viral transcription/replication (3 to 9 h p.i.) and release (6 to 9 h p.i.) [6]. In our present study, we further investigate the effect of rhein, a major ingredient of rhubarb, and find that rhein can significantly inhibit IAV adsorption and replication, but cannot significantly inactivate IAV and has no significant influence on cells before infection.

IAV infection can cause severe oxidative stress, and inhibition of oxidative stress is now believed to be a novel target for pharmacologic treatment of IAV infection [35–37]. It has been reported that GSH can decrease viral titer in both lung and trachea homogenates in BALB/c mice [38]. Recombinant human CAT, SOD and GSH-PX can reduce IAV-induced lung inflammation and IAV titer in lungs of mice [39–41]. Rhein possesses anti-oxidative activity and can significantly increase the viability of H_2_O_2_-injured human umbilical vein endothelial cells with decreased MDA and LDH production and increased SOD and GSH-PX activity [27]. Rhein can decrease the expressions of NADPH oxidase subunits p22^phox^ and gp91^phox^ [24]. In the present study, we find that rhein can significantly decrease the production of MDA, NO, and ROS, increase the level of GSH and the activities of T-SOD, GR, CAT, and GSH-PX after IAV infection.

Oxidative stress and activation of TLRs can mutually promote and develop together. Oxidative stress can serve as a potential activator of TLRs [42]. In turn, activations of TLR2, TLR3, and TLR4 can increase lipid and protein oxidation levels [43]. Activations of TLR2 and TLR4 can increase NADPH oxidase expression and activity [44, 45]. Oxidative stress also can activate PI3K/Akt, MAPK, and NF-κB signaling pathways [46, 47]. IAV-induced oxidative stress and activations of TLRs, MAPK, and NF-κB signal pathways are essential for efficient IAV replication [13–19]. Blockade of IAV-induced Raf/MEK/ERK MAPK signal cascade can retard IAV ribonucleoprotein (RNP, consisting of PB2, PB1, PA, NP, and vRNA) export and reduce IAV titers [17]. JNK inhibitors SP600125 and AS601245 can decrease IAV amplification by suppressing viral protein and RNA synthesis [18]. NF-κB inhibitors can specifically diminish IAV vRNA transcription from the cRNA promoter [13]. Activation of p38 is also needed for IAV replication [19]. Beside these pathways, activation of PI3K/Akt pathway is also indispensable for IAV replication. Inhibition of Akt kinase activity can suppress the entry and replication of IAV and prevent IAV infection [15,16]. Many previous studies have reported that rhein can inhibit the activations of TLRs, MAPK, NF-κB, and PI3K/Akt pathways. Rhein can reduce LPS-induced TLR4 expression and inhibit NF-κB activation in colon tissue [25]. Rhein can significantly decrease the expressions of TLR4 and MyD88 and inhibit the activations of NF-κB and JNK MAPK pathways in high-fat diet-induced obese male mice [26]. Rhein can significantly inhibit the activations of PI3K/Akt, p-ERK, NF-κB and COX-2 pathways [48], and significantly suppress the phosphorylation of ERK, p38 MAPK and activation of NF-κB in human nasopharyngeal carcinoma cells [49]. In our present study, we find that rhein can significantly decrease IAV-induced expressions of TLR2, TLR3, and TLR4, and suppress IAV-induced activations of Akt, p38/JNK MAPK and NF-κB pathways.

Excessive production of inflammatory cytokines is an essential pathogenic factor that leads to ALI and ARDS. Our study has showed that rhein can significantly reduce the expressions of IL-1β, IL-6, IL-8, and TNF-α after IAV infection. Moreover, excessive production of MMPs, which can degrade collagen and other components of extracellular matrix, is also an essential pathogenic factor of ALI and ARDS. IAV infection can increase the expressions or activities of MMP-2, MMP-9, and TIMP-1 [50, 51]. In our study, we also find that rhein can significantly inhibit IAV-induced production of MMP2, MMP3, and MMP9. In addition, it has been reported that activation of TLRs plays an important role in linking oxidative stress to inflammation [52]. So, we speculate that the inhibition of rhein on the production of inflammatory cytokines and MMPs may be due to the inhibition of rhein on IAV-induced activations of TLRs, Akt, p38, JNK MAPK, and NF-κB pathways. In fact, rhein can inhibit the expressions of MMP-1, MMP-3, MMP-9, and MMP-13 by suppressing the phosphorylation of ERK, p38, JNK MAPK and the transcription activities of NF-κB and AP-1 [49, 53].

In the present study, we have also investigated the counteracting effects of different agonists using two methods. One is the SRB method utilizing CPE reduction to determine the finally remained cell viability after IAV infection and drug treatments. The other method is the qRT-PCR assay to determine the transcription of IAV genome. In fact, the degree of virus-induced CPE is not completely determined by virus replication or virus titer, and drugs that can inhibit virus-induced CPE are uncertain to be able to inhibit viral transcription. In our study, we find that oxidant H_2_O_2_ and agonists of TLR2, TLR4, Akt, p38, JNK, and IKK/NF-κB can significantly antagonize the inhibition of rhein on IAV-induced CPE, but the qRT-PCR assay shows that TLR2 agonist cannot antagonize the effect of rhein on IAV transcription.

Finally, our *in vivo* test shows that rhein can significantly improve the survival rate of mice and lung histopathological changes, suppress the lung index and the production of lung inflammatory cytokines, and at the high dose, rhein can reduce pulmonary viral load. Similar researches have showed that rhein can prevent LPS-induced acute kidney injury by inhibiting NF-κB activities [54] and inhibit LPS-induced intestinal injury during sepsis by blocking TLR4- NF-κB pathway [55].

Additionally, our result also shows that rhein can inhibit IAV adsorption. As for the mechanism, we have not investigated in the present study, and we speculate that the inhibition of rhein on viral adsorption may be through ① interfering IAV hemagglutinin that is an important protein of IAV for viral adsorption, or ② interfering lipid raft, which functions as a platform for formation of viral membrane fusion.

## Conclusion

**Fig 8:**
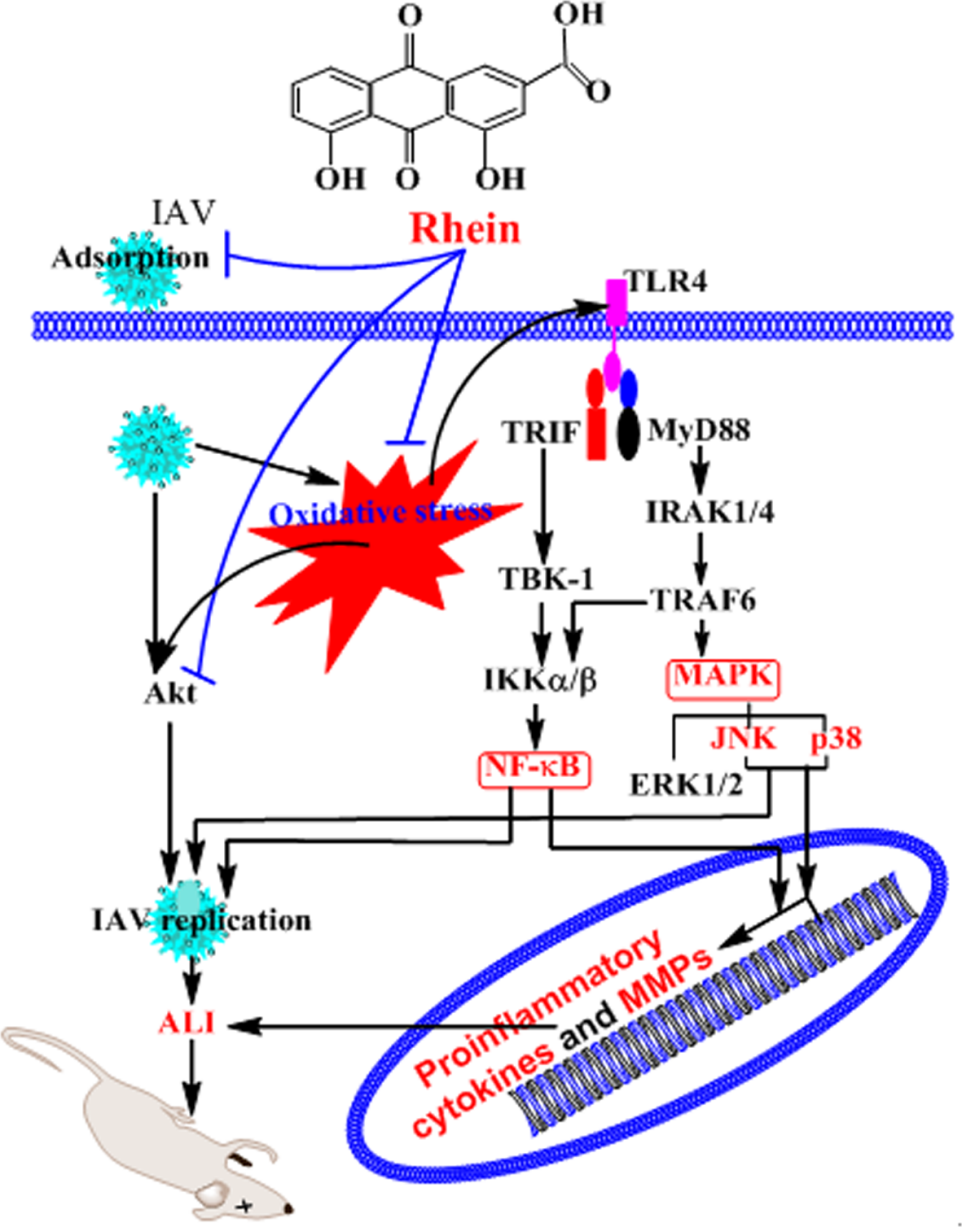
Schematic diagram of rhein on inhibition of IAV infection and IAV-mediated ALI. Rhein can inhibit IAV adsorption and replication. Rhein suppresses IAV replication by inhibiting IAV-mediated oxidative stress and activations of TLR4, Akt, p38, JNK MAPK, and NF-κB signal pathways. Meanwhile, inhibition of these signal pathways further reduces the production of inflammatory cytokines and MMPs and finally decreases IAV-induced ALI.

Through *in vitro* and *in vivo* tests, we have found that rhein can inhibit IAV adsorption and replication, the mechanism of action of rhein to inhibit IAV replication may be related to its ability to inhibit IAV-mediated oxidative stress and IAV-induced activations of TLR4, Akt, p38, JNK MAPK, and NF-κB signal pathways. Inhibition of these signal pathways may simultaneously reduce the production of inflammatory cytokines and MMPs, and finally leading to suppress IAV-induced ALI (Fig 8).

## Supporting information

S1 Table This is the S1 Table title. This is the S1 Table legend.
(PDF)
