# Supplementary material for: Anti-influenza A virus activity of rhein through regulating oxidative stress, TLR4, Akt, MAPK, and NF-κB signal pathways
Source: PLoS One. 2018 Jan 31;13(1):e0191793. doi: 10.1371/journal.pone.0191793 (PMC5791991; doi:10.1371/journal.pone.0191793)
Supplement: S1 Table — This is the S1 Table legend. (PDF) [file pone.0191793.s001.pdf]

S1 Table. The sequences of primers used in qRT-PCR assay.

genes	Forward (5'→3')	Reverse (5'→3')
Human:		
β-actin	CCTGACTGACTACCTCATGAAG	GACGTAGCACAGCTT CTCCTTA
IL-1β	AGCTACGAATCTCCGACCAC	CGTTATCCCATGTGTCGAAGAA
IL-6	AGCCACTCACCTCTTCAGAAC	ACATGTCTCCTTTCTCAGGGC
IL-8	AGGAGTGCTAAAGAACTTCGA	TGA ATTCTCAGCCCTCTTCAA
IL-10	CCCTGTGAAAACAAGAGCAA	TGGTCAGGCTTGGAATGGAA
TNF-α	CCTCTCTCTAATCAGCCCTCTG	GAGGACCTGGGAGTAGATGAG
MMP-2	ATTCCGCTTCCAGGGCACA	GGTCTCAGGGCAGAAGCCATAC
MMP-3	ATTCCATGGAGCCAGGCTTTC	CATTTGGGTCAAACTCCAACTGTG
MMP-9	ACGCACGACGTCTTCCAGTA	CCACCTGGTTCAACTCACTCC
MMP-13	TTGATGATGATGAAACCTGGACAAG	TTGCCGGTGTAGGTGTAGATAGGAA
TIMP-1	AATTCCGACCTCGTCATCAG	TGCAGTTTTCCAGCAATGAG
IAV M	GGACTGCAGCGTAGACGCTT	CATCCTGTTGTATATGAGGCCCAT
Mouse:		
β-actin	TGGAATCCTGTGGCATCCATGAAAC	TAAAACGCAGCTCAGTAACAGTCCG
IL-1β	AAGGAGAACCAAGCACGACAAAA	TGGGGAACTCTGCAGACTCAAACT
IL-6	GAGGATACCACTCCCAACAGACC	AAGTGATCATCGTTGTTCATACA
IL-8	CAA ACC TTT CCA CCC CAA AT	ATT GCA TCT GGC AAC CCT AC
IL-10	CCCAGAAATCAAGGAGCATT	TCACTCTTCACCTGCTCCAC
TNF-α	CCAAAGGGATGAGAAGTTCC	CTCCACTTGGTGGTTTGCTA
